# NADPH oxidase 4 regulates anoikis resistance of gastric cancer cells through the generation of reactive oxygen species and the induction of EGFR

**DOI:** 10.1038/s41419-018-0953-7

**Published:** 2018-09-20

**Authors:** Shangce Du, Ji Miao, Zhouting Zhu, En Xu, Linsen Shi, Shichao Ai, Feng Wang, Xing Kang, Hong Chen, Xiaofeng Lu, Wenxian Guan, Xuefeng Xia

**Affiliations:** 1 Department of General Surgery, Drum Tower Clinical Medical College of Nanjing Medical University, 321 Zhongshan Road, 210008 Nanjing, Jiangsu P. R. China; 2 Department of General Surgery, Nanjing Drum Tower Hospital, The Affiliated Hospital of Nanjing University Medical School, 321 Zhongshan Road, 210008 Nanjing, Jiangsu P. R. China

## Abstract

Anoikis is a type of programmed cell death induced by detachment from the extracellular matrix. In cancer cells, anoikis resistance is essential for cancer cell survival in blood circulation and distant metastasis. However, the mechanisms behind anoikis resistance of gastric cancer remain largely unknown. Herein, we demonstrate that NADPH oxidase 4 (NOX4) expression and reactive oxygen species (ROS) generation are upregulated in suspension gastric cell cultures compared with adherent cultures. Silencing of NOX4 decreases ROS generation and downregulates EGFR, sensitizing cells to anoikis. NOX4 overexpression upregulates ROS and EGFR levels and promotes anoikis resistance. NOX4 depletion inhibits gastric cancer survival in blood circulation and attenuates distant metastasis. NOX4 expression is correlated with EGFR expression in patients. In conclusion, induction of NOX4 expression by detachment promotes anoikis resistance of gastric cancer through ROS generation and downstream upregulation of EGFR, which is critical for the metastatic progression of gastric cancer.

## Introduction

Gastric cancer (GC) is one of the most common malignancies and the third most common cause of cancer deaths worldwide^1^. The prognosis for patients with GC is very poor and the 5-year survival rate is less than 30%^2^. It is mainly metastasis that accounts for the high mortality rate^3^. As a programmed cell death triggered by detachment from the extracellular matrix (ECM), anoikis prevents detached cell growth and re-attachment to new matrices in ectopic locations, preventing colonization of distant organs^4^. In contrast to healthy cells, cancer cells can evade anoikis, which contributes to tumor progression and metastasis^5^.

Redox homeostasis is essential for the regulation of cellular metabolism, survival, and growth. ROS are essential to overcome apoptosis through modulation of multiple signaling cascades related to proliferation, angiogenesis, and survival^6,7^. Moreover, ROS can stimulate many metastasis-related signals, triggering cancer cell invasion through intravasation and extravasation into distant sites^8^. Many sources of ROS in cells have come to light, including NADPH oxidase (NOX) and the mitochondrial electron transfer chain. NOX-derived ROS have been identified as the main source of oxidative stress that promotes carcinogenesis and metastasis^9^. NOX4 is one of seven NOX family members that transports electrons from NADPH to oxygen, generating hydrogen peroxide (H_2_O_2_) and the ROS superoxide anion (O_2_^−^)^10^.

In GC tissue, expression of NOX4 is significantly higher than in adjacent healthy tissue^11^. Furthermore, in several cancer cell lines, NOX4 has been shown to be involved in regulation of cell proliferation^12^, invasion^13^, and migration^14^, as well as epithelial-mesenchymal transition (EMT) and invadopodia formation^15^.

Epidermal growth factor receptor (EGFR) is a receptor tyrosine kinase^16^. Overexpression of EGFR is detected in 27–44% of gastric cancer cases and is associated with a poor prognosis^17^. Phosphorylation of EGFR promotes cell survival, proliferation, differentiation, and migration, and is implicated in the progression of various malignancies, including gastric cancer^17,18^. Overexpression of EGFR is involved in anoikis resistance through downregulation of the proapoptotic protein Bim^19^. Furthermore, upon detachment from the ECM, EGFR is bound and inhibited by CCN family protein 2 (CCN2), promoting anoikis by enhancing the expression of apoptosis-associated protein kinases^20^. Expression and activation of EGFR, therefore, plays a key role in anoikis resistance of cancer cells.

In this study, we demonstrate that detachment from the ECM triggers NOX4 upregulation, which increases ROS expression and downstream upregulation of EGFR. During detachment, downregulation of NOX4 by siRNA enhances EGFR downregulation, attenuating GC cell resistance to anoikis. Upregulation of NOX4 using an expression plasmid impairs EGFR downregulation, promoting resistance to anoikis. In vivo, invasion and re-attachment to distant organs by GC cells was inhibited by knockdown of NOX4. Furthermore, expression of NOX4 is positively correlated with expression of EGFR in GC patients.

## Results

### GC cells are more anoikis-resistant than normal gastric epithelial cells

It has been proved that cancer cells are less sensitive to anoikis compared with normal cells when unattached from the ECM^21^. As the suspension culture progressed, the number of normal gastric epithelial cell line, GES-1 decreased while the number of GC cell lines, MKN-45 and AGS increased, although their growth rate was extremely slow (Supplementary Fig. 1A). The rate of apoptosis in the GES-1 suspension culture was significantly higher than in the adherent culture. In the GC cancer cells, however, differences in the rate of apoptosis in adherent and suspension cultures were not as remarkable (Supplementary Fig. 1B). Compared with GES-1, MKN-45 and AGS cells aggregated to form larger colonies at a faster rate during suspension (Supplementary Fig. 1C). In addition, the number of aggregated MKN-45 and AGS cells was significantly higher than GES-1 cells (Supplementary Fig. 1D). In suspension, cells forming multicellular aggregates are more anoikis-resistant than single cell suspensions^22^. The activation of caspase-3, which presents as cleaved caspase-3, was enhanced in GES-1, MKN-45, and AGS suspension cultures as compared to adherent cultures, indicating that cells underwent varying degrees of apoptosis. The enhanced level of caspase-3 activation in suspension cultures was more prominent in GES-1 cells than in MKN-45 and AGS cells (Supplementary Fig. 1E). Together, these data suggest that GC cells are more anoikis-resistant than normal gastric epithelial cells.

### NOX4 upregulation and ROS generation are involved in the anoikis resistance of GC cells

**Fig. 1:**
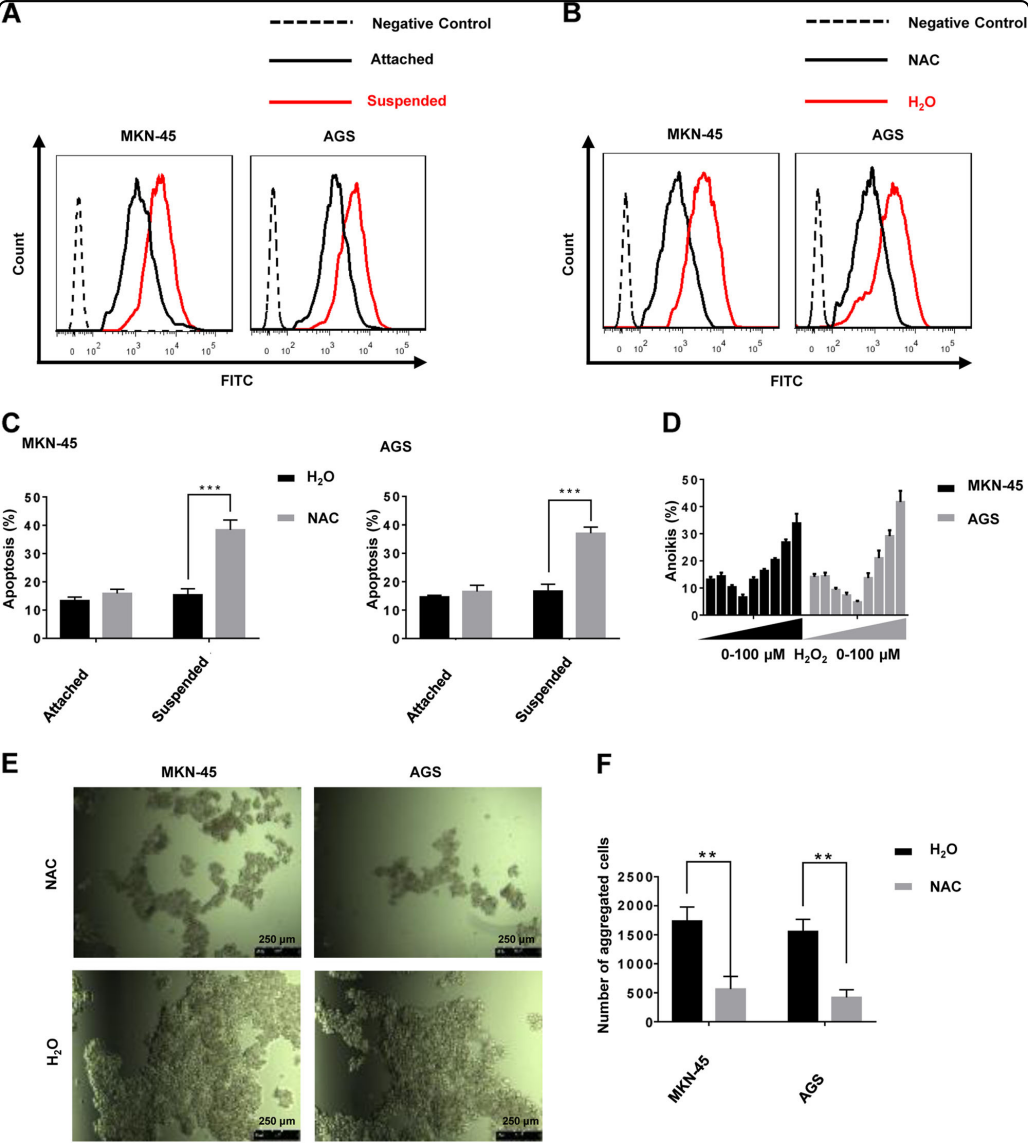
ROS generation is involved in the anoikis resistance of gastric cancer cells. **a** ROS generation by adherent or suspension MKN-45 and AGS cultures was measured by flow cytometry using carboxy-H2DCFDA. The solution that contains GC cells without treatment using ROS-sensitive substrate H2DCFDA was used as the negative control. **b** ROS generation by MKN-45 and AGS suspension cultures treated with H_2_O or NAC was measured by flow cytometry using carboxy-H2DCFDA. **c** Anoikis rates of MKN-45 and AGS cells treated with H_2_O or NAC in suspension conditions for 24 h were measured by flow cytometry using Annexin V-FITC. ***P < 0.001. **d** MKN-45 and AGS cells were treated with exogenous H_2_O_2_ (0, 0.1, 0.5, 1, 2, 5, 10, 50, 100 μM), and the rate of anoikis detected by flow cytometry using Annexin V-FITC. **e** Cellular morphology of MKN-45 and AGS suspension cells treated with H_2_O or NAC captured by microphotography after 24 h of culture (magnification ×100). **f** The average number of aggregated MKN-45 and AGS suspension cells treated with H_2_O or NAC in each field was measured after 24 h of culturing. **P < 0.01. All experiments were performed three independent times

**Fig. 2:**
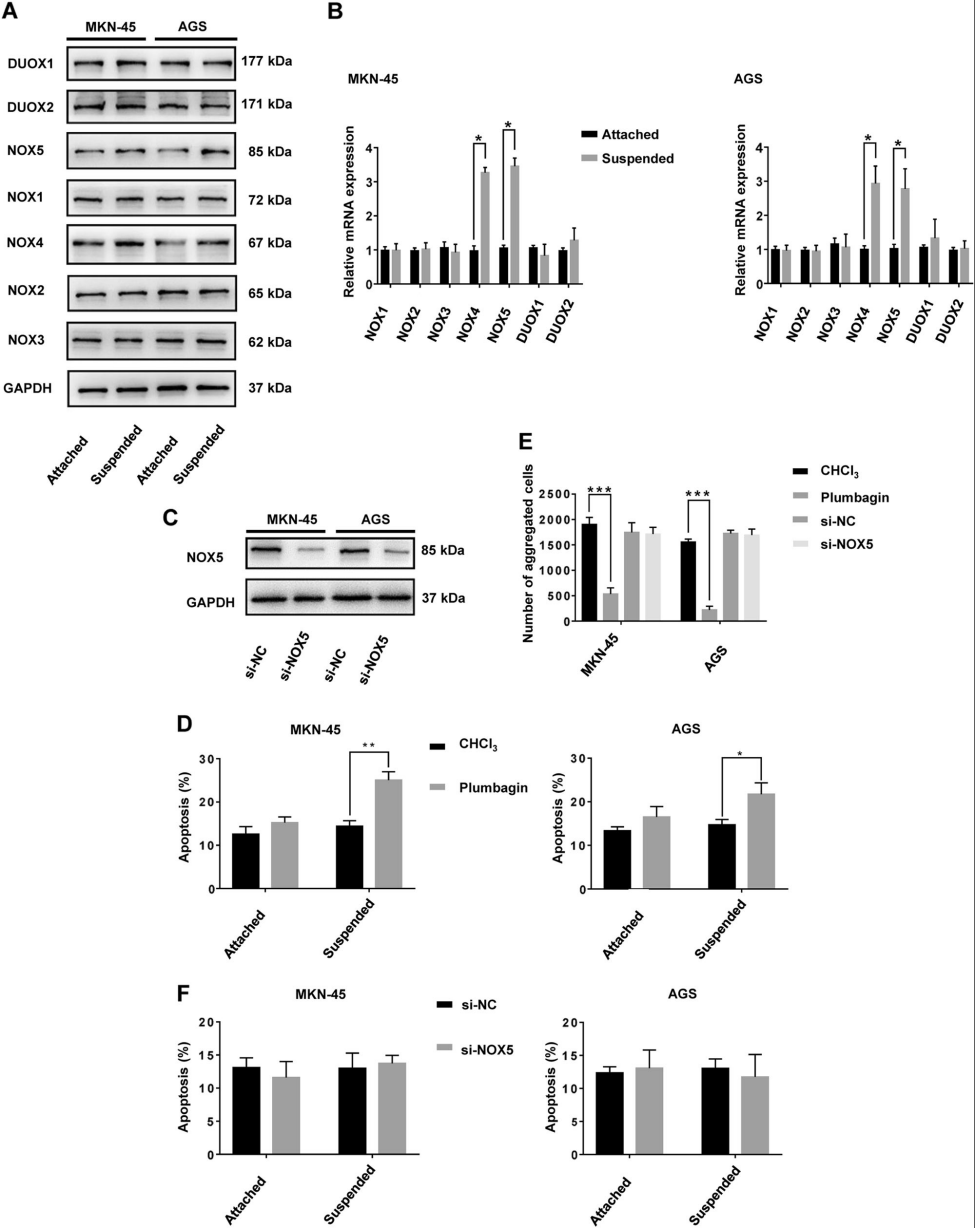
NOX4, and not NOX5, is involved in regulation of anoikis resistance. MKN-45 and AGS cells were cultured in attached or suspended conditions for 24 h followed by total mRNA and protein extraction. **a** The expression of NOX1-5 and DUOX1-2 was measured by immunoblotting with the indicated antibodies. GAPDH served as a loading control. **b** The relative mRNA expression of NOX1-5 and DUOX1-2 was measured by RT-qPCR. *P < 0.05. All experiments were performed three independent times. **c** MKN-45 and AGS cells were transfected with siRNA-NOX5 and siRNA-Negative Control (NC) for 24 h, and the expression of NOX5 protein detected by immunoblotting using the indicated antibodies. GAPDH served as a loading control. **d** MKN-45 and AGS cells cultured in attached or suspended conditions were treated with plumbagin (10 μM), a specific NOX4 inhibitor, or CHCl_3_, a solvent of plumbagin, and the apoptosis rate measured by flow cytometry using Annexin V-FITC. *P < 0.05, **P < 0.01. **e** The average number of aggregates of MKN-45 and AGS suspension cells treated with plumbagin or CHCl_3_ or with si-NC or si-NOX5 in each field was measured after 24 h of culture. ***P *<* 0.001. **f** MKN-45 and AGS cells were transfected with siRNA-NOX5 or siRNA-negative Control for 24 h, and the apoptosis rate measured by flow cytometry using Annexin V-FITC. All experiments were performed in triplicate

Reactive oxygen metabolites are essential for the maintenance of cancer cell metabolism, genomic instability, proliferation, and angiogenesis^7^. Furthermore, ROS as signaling molecules, can promote cell survival over apoptosis^23^. ROS levels in the suspended cells increased compared with those in attached cells (Fig. 1a). ROS levels decreased by treatment with *N*-acetylcysteine (NAC), a ROS scavenger (Fig. 1b), which was accompanied by a remarkable increase in the rate of anoikis in cells in suspended conditions (Fig. 1c). In contrast, treatment with NAC did not significantly increase the rate of apoptosis of GC cells grown in attached conditions (Fig. 1c). Interestingly, the addition of H_2_O_2_ in small doses (0–1 μM for MKN-45, 0–2 μM for AGS) decreased the rate of anoikis in GC suspension cultures. However, as H_2_O_2_ concentrations increased (>1 μM for MKN-45, >2 μM for AGS) the rate of anoikis increased (Fig. 1d). In addition, GC cells formed smaller aggregates at a slower rate, and the number of aggregates decreased during NAC treatment of suspension cultures (Fig. 1e, f). NOX-derived ROS have been shown to be the main source of oxidative stress in cells, triggering the initiation and progression of cancer^9^. Therefore, we compared mRNA and protein levels of the seven NOX family subtypes in GC cell adherent or suspension cultures. Only NOX4 and NOX5 expression was significantly different between the two conditions at the levels of transcription and translation (Fig. 2a, b). To determine the contribution of NOX4 and NOX5 to anoikis resistance of GC cells, we performed siRNA knockdown of NOX5. Knockdown efficiency was verified by western blot (Fig. 2c). It was demonstrated that plumbagin was a specific inhibitor for NOX4 but not for other NADPH oxidases (NOX1-3,5, DUOX1-2)^24^. Mechanically, plumbagin could directly inhibit NOX4 activity and nuclear NOX4 binding network, such as the binding of NOX4 with nucleoskeleton components and phospho-ERK^25^. Under the treatment with plumbagin, the anoikis rate of GC cells increased significantly in suspension cultures, while the rate remained almost unchanged in adherent cultures (Fig. 2d). Treatment with plumbagin inhibited aggregate formation both in terms of volume, rate and number of GC cell aggregates in suspension culture (Supplementary Fig. 2A and Fig. 2E). However, knockdown of NOX5 had little effect on the apoptosis rate of GC cells either in attached or suspended conditions (Fig. 2f). Additionally, knocking down NOX5 hardly inhibited any aspect of aggregate formation of GC cells in suspension culture (Supplementary Fig. 2B and Fig. 2E).

**Fig. 3:**
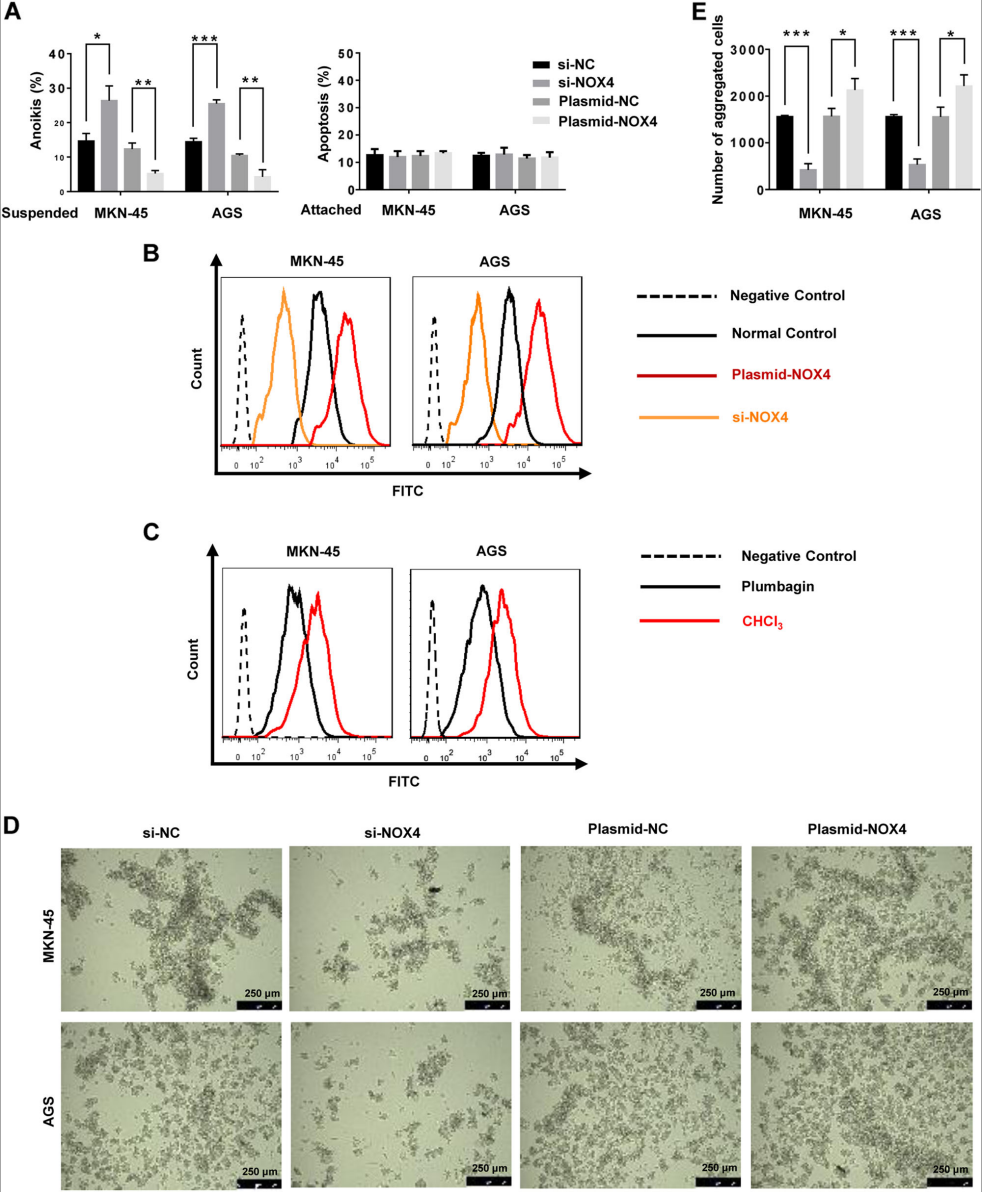
Effects of NOX4 on anoikis resistance and ROS generation. MKN-45 and AGS cells were transfected with siRNA-NOX4, siRNA-Negative Control, plasmid- NOX4 or plasmid-Negative Control for 24 h. **a** The apoptosis rate of suspended or adherent GC cells detected by flow cytometry using Annexin V-FITC. *P < 0.05, **P < 0.01, ***P < 0.001. **b** ROS generation by MKN-45 and AGS suspension cells as measured by flow cytometry using carboxy-H2DCFDA. **c** ROS generation by MKN-45 and AGS suspension cells treated with plumbagin or CHCl_3_ as measured by flow cytometry using carboxy-H2DCFDA. **d** Morphology of MKN-45 and AGS suspension cultures as captured by microphotography (magnification ×100). **e** The average number of aggregated MKN-45 and AGS cells in suspension cultures in each field was measured after 24 h of culture. *P < 0.05, ***P < 0.001. All experiments were performed three independent times

To explore the involvement of NOX4 in anoikis resistance, NOX4 was depleted or overexpressed by siRNA or plasmid transfection, respectively. The knockdown and overexpression efficiencies were verified at the levels of transcription and translation. Overexpression of NOX4 moderately decreased the rate of anoikis in suspension GC cell cultures and increased ROS levels, while depletion of NOX4 notably increased the rate of anoikis and led to a decrease in ROS levels (Fig. 3a, b). Treatment with the NOX4 inhibitor plumbagin similarly decreased the generation of ROS (Fig. 3c). Interestingly, neither overexpression nor depletion of NOX4 affected the rate of apoptosis of adherent GC cells, indicating the regulation of apoptosis in GC cells by NOX4 selectively occurs in suspension culture (Fig. 3a). Overexpression of NOX4 promoted the formation of aggregates in terms of volume and rate as well as the number of GC cell aggregates during suspension (Fig. 3d, e). In contrast, depletion of NOX4 had the opposite effects (Fig. 3d, e).

### NOX4 positively regulates the expression of EGFR

**Fig. 4:**
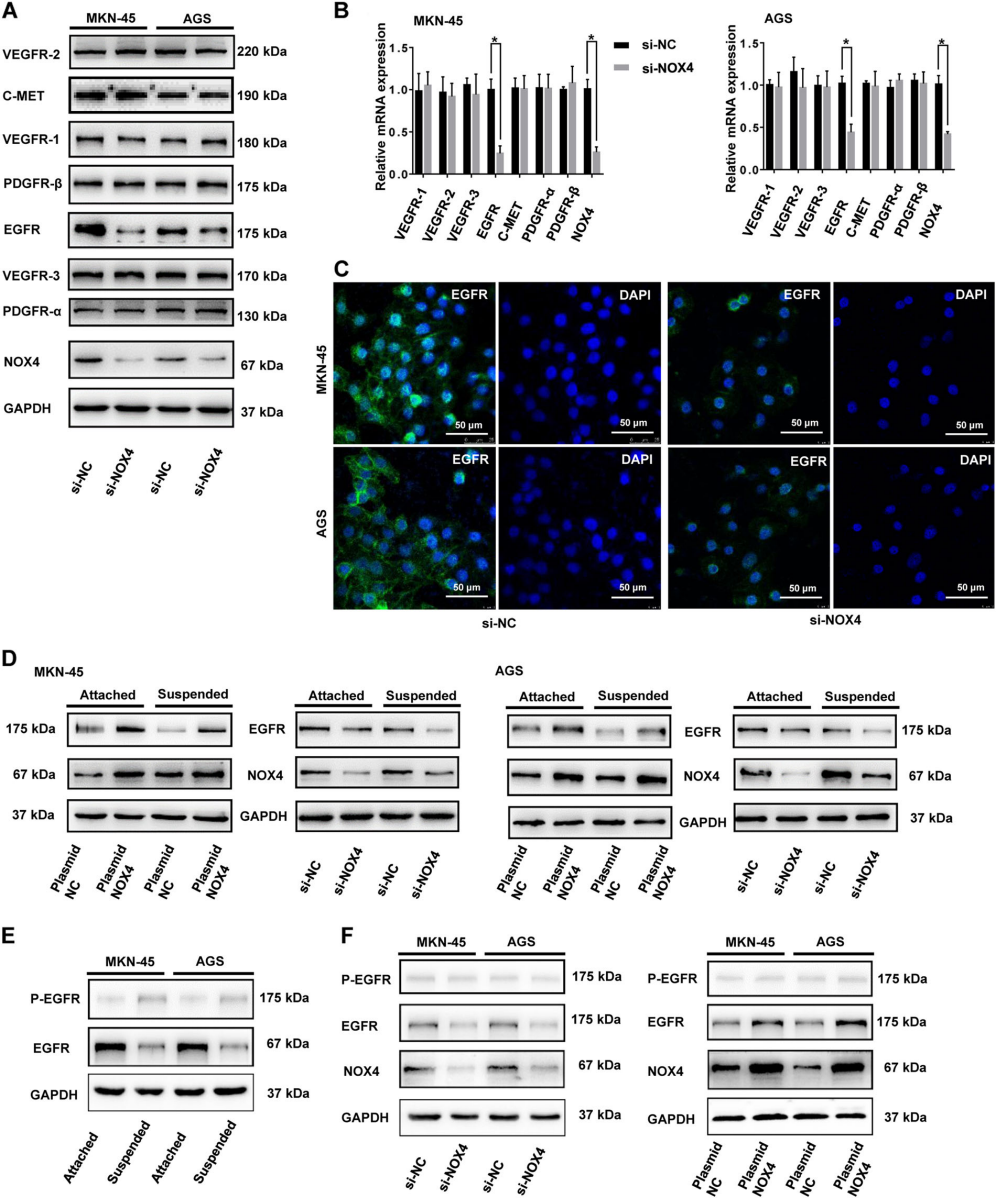
NOX4 positively regulates the expression of EGFR. MKN-45 and AGS cells were transfected with siRNA-NOX4 or siRNA-Negative Control for 24 h. **a** The expression of selected growth factor receptors was detected by immunoblotting with the indicated antibodies. **b** Relative mRNA expression levels were measured by RT-qPCR. *P < 0.05. **c** EGFR expression was detached by immunofluorescence (magnification ×500). **d** MKN-45 and AGS cells were transfected with siRNA-NOX4, siRNA-Negative Control, plasmid-NOX4 or plasmid-Negative Control for 24 h. Expression of NOX4 and EGFR was detected by immunoblotting with the indicated antibodies. **e** MKN-45 and AGS cells were cultured in adherent or suspended conditions for 24 h, expression of EGFR and p-EGFR was detected by immunoblotting with the indicated antibodies. **f** MKN-45 and AGS cells were transfected with siRNA-NOX4, siRNA-Negative Control, plasmid-NOX4 or plasmid-Negative Control for 24 h. Expression of EGFR and p-EGFR was detected by immunoblotting with the indicated antibodies. All experiments were performed in triplicate

It has previously been proved that maintenance of growth factor receptors are involved in cell survival^26–28^, as well as in resistance to apoptosis. We therefore compared expression of VEGFR-1, VEGFR-2, VEGFR-3, EGFR, PDGFR-α, PDGFR-β, and C-Met in GC cells and si-NOX4-transfected GC cells in suspension conditions. Of these growth factors, only EGFR expression was distinctly regulated by NOX4 at the levels of transcription and translation (Fig. 4a, b). In GC cells treated with si-NC, EGFR was mainly localized to cytoplasm and the plasma membrane, while in NOX4 knockdown cells the plasma membrane-localized EGFR and cytoplasmic EGFR expression were very weak (Fig. 4c). Overexpression of NOX4 upregulated EGFR expression in both adherent and suspension cultures. Interestingly, the regulatory effects of NOX4 on EGFR in suspension were more notable than in attachment cultures (Fig. 4d). Moreover, EGFR expression of GC cells was lower in suspension compared with adherent conditions, whereas p-EGFR expression was enhanced in suspended GC cells as compared to adherent ones (Fig. 4e). This phenomenon indicated that EGFR activation might also be involved in the regulation of anoikis resistance of GC cells. However, the expression of p-EGFR in suspended GC cells was very weak. What’s more, either depletion or overexpression of NOX4 could hardly regulate p-EGFR levels in suspended GC cells (Fig. 4f). Hence, NOX4 positively regulates the expression of EGFR but not EGFR activation in suspended GC cells.

### NOX4 promotes anoikis resistance through ROS-dependent upregulation of EGFR

**Fig. 5:**
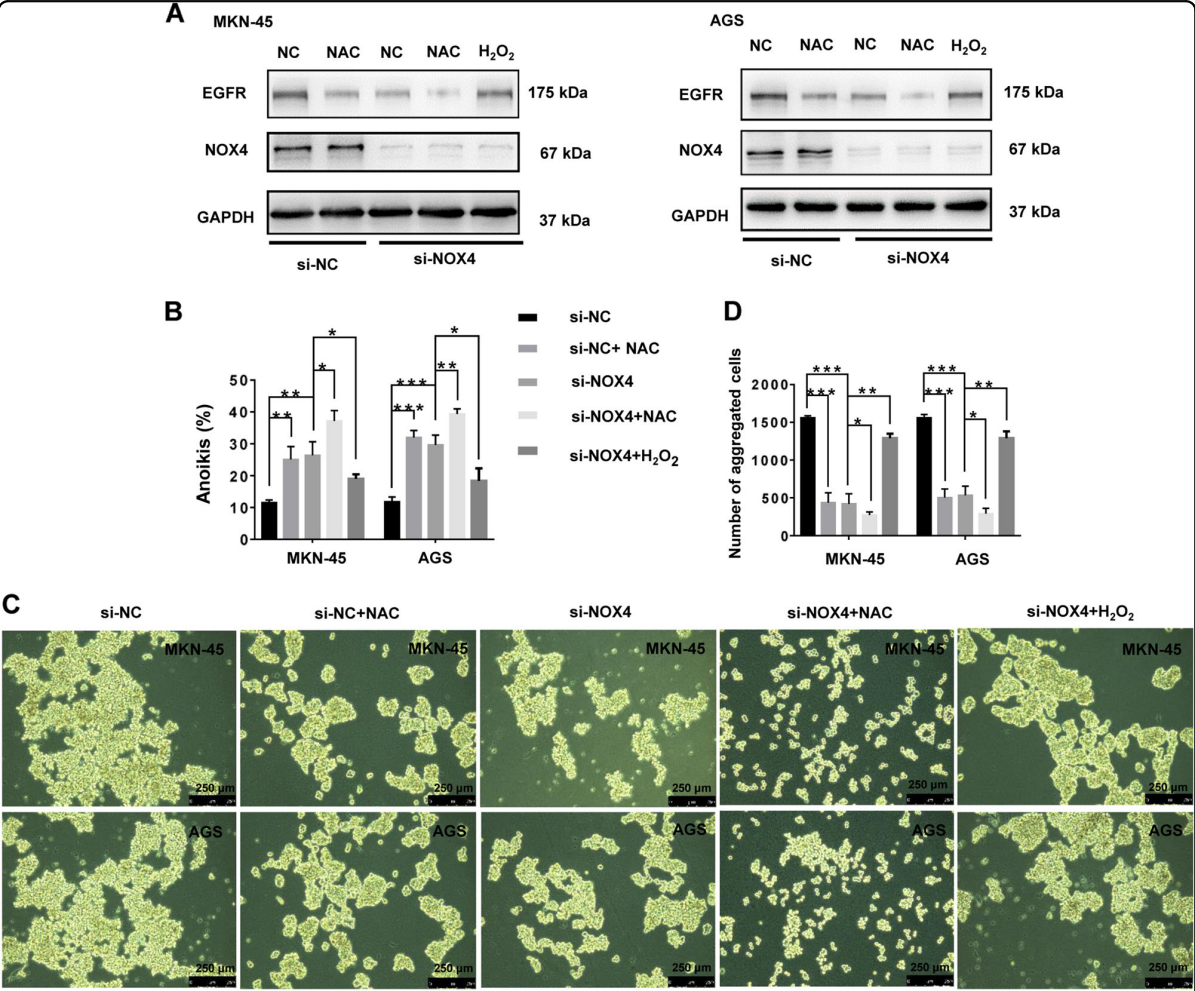
NOX4 regulates anoikis resistance and EGFR expression via ROS generation. MKN-45 and AGS cells were treated with si-NC, NAC, si-NOX4, si-NOX4 + NAC or si-NOX4 + H_2_O_2_ (1 μM). (**a**) The expression of NOX4 and EGFR was detected by immunoblotting with the indicated antibodies. (**b**) The rate of anoikis in MKN-45 and AGS cells was measured by flow cytometry using Annexin V-FITC. *P < 0.05, **P < 0.01, ***P < 0.001. (**c**) Cellular morphology of MKN-45 and AGS cells in suspension conditions captured by microphotography (magnification x100). (**d**) The average aggregated number of MKN-45 and AGS suspension cells in each field was measured after 24 h of culturing. *P *<* 0.05, ***P < 0.001. All experiments were performed three independent times

EGFR expression in GC suspension cells was downregulated following treatment with the ROS inhibitor NAC, similar to the effect of treatment with si-NOX4. Furthermore, the downregulation of EGFR in GC cells observed with knockdown of NOX4 was partly blocked by increasing the levels of ROS by H_2_O_2_ treatment (Fig. 5a). In contrast, the upregulation of EGFR by overexpression of NOX4 was somewhat decreased through the scavenging of ROS by NAC (Supplementary Fig. 3A). Therefore, NOX4 regulates EGFR through ROS generation.

The proapoptotic effects triggered by knocking down NOX4 were blocked by H_2_O_2_ treatment (Fig. 5b)_._ Treatment also upregulated EGFR and promoted aggregation in si-NOX4-transfected GC cells (Fig. 5a, c, d). Interestingly, treatment with NAC and si-NOX4 had an additive proapoptotic effect on GC suspension cells (Fig. 5a–d). Conversely, the antiapoptotic effects triggered by overexpression of NOX4 were blocked by the depletion of ROS (Supplementary Fig. 3B), which was accompanied by the downregulation of EGFR as well as the inhibition of aggregation of plasmid-NOX4-transfected GC cells (Supplementary Fig. 3A, C and D). We next focused on EGFR expression. Knocking down EGFR clearly promoted anoikis of GC cells in suspension (Supplementary Fig. 4A and B), inhibiting the rate of formation and volume of GC cell aggregates (Supplementary Fig. 4C and D). Furthermore, the antiapoptotic effects observed following upregulation of NOX4 in GC suspension cells were reduced with knockdown of EGFR (Supplementary Fig. 4A, B, C and D). Therefore, the NOX4−ROS−EGFR axis plays an essential role in the anoikis resistance of GC cells.

### The biological characteristics of anoikis-resistant gastric cancer cells

**Fig. 6:**
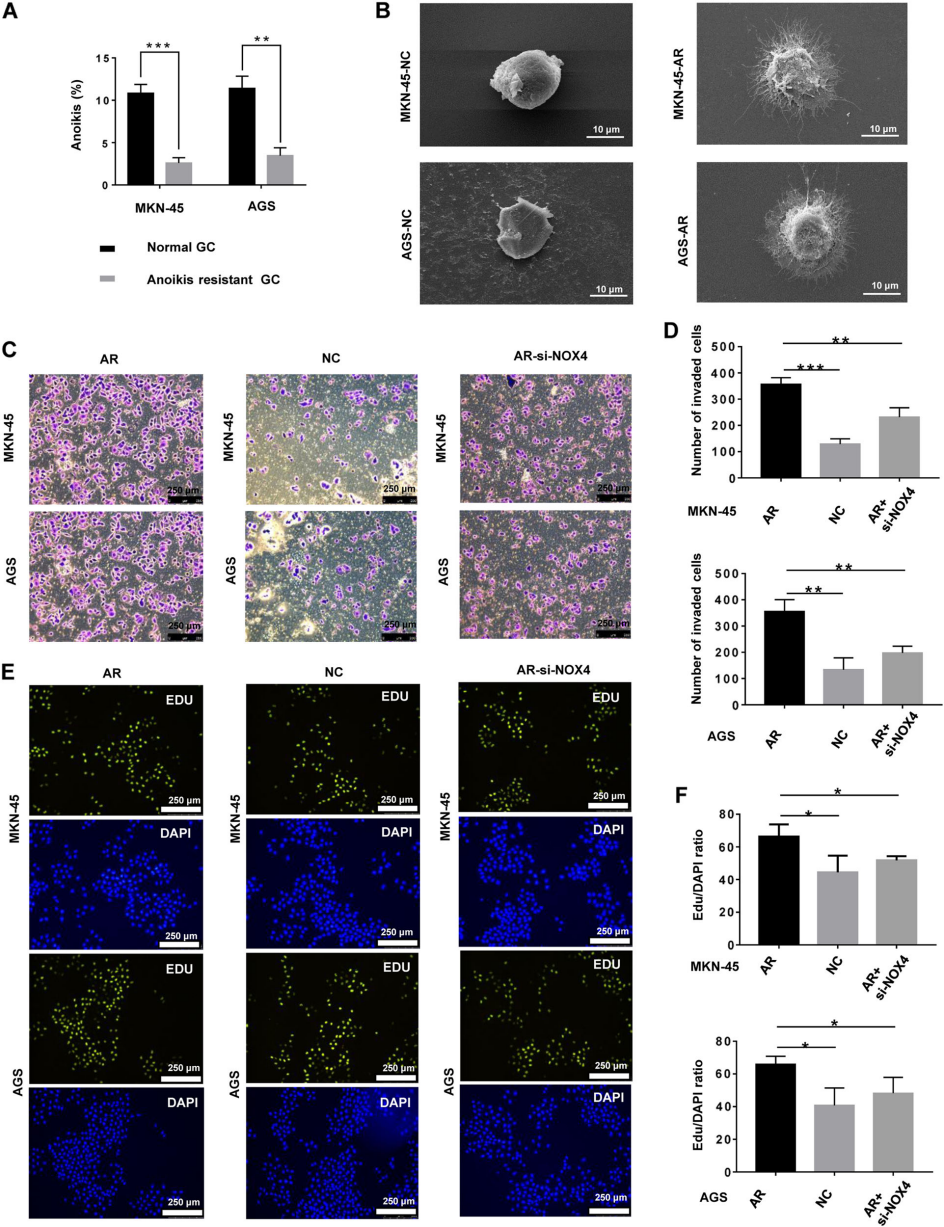
The biological characteristics of anoikis-resistant gastric cancer cells. **a** The rate of anoikis of MKN-45 and MKN-45-AR cells was measured by flow cytometry using Annexin V-FITC. **P *<* 0.01, ***P < 0.001. **b** Scanning electron micrograph of gastric cancer cells and anoikis-resistant gastric cancer cells (magnification ×2000). **c** Invasion of gastric cancer cells, anoikis-resistant gastric cancer cells, and anoikis-resistant gastric cancer cells with NOX4 knockdown was assessed by a Matrigel invasion assay system (magnification x100). **d** The average number of invaded cells in each field was measured. **P *<* 0.01, ***P < 0.001. **e** Proliferation of gastric cancer cells, anoikis-resistant gastric cancer cells, and anoikis-resistant gastric cancer cells with NOX4 knockdown was assessed by EdU incorporation. **f** The average Edu/DAPI ratio of cells in each field was measured. *P *<* 0.05. All experiments were performed three independent times (magnification ×100)

Some gastric cancer cells undergo anoikis upon detachment from the ECM, while GC cells that are resistant to anoikis survive. To determine the biological characteristics of GC cells that are insensitive to anoikis, we generated anoikis-resistant GC cells (GC^AR^) by continuous culture in suspension for 60 days. The anoikis rate of GC^AR^ cells was lower than ordinary GC cells (Fig. 6a). Furthermore, GC^AR^ cells underwent significant morphological transformations. Compared with ordinary GC cells, GC^AR^ cells branched out multiple invadopodia (Fig. 6b). The presence of invadopodia is probably associated with advanced invasion and migration of cancer cells^29^. Moreover, compared with ordinary GC cells, GC^AR^ cells showed increased proliferation and invasion (Fig. 6c–f), suggesting that GC^AR^ cells had stronger metastasis ability. Moreover, knockdown of NOX4 effectively inhibited the proliferation and invasion of GC^AR^ cells (Fig. 6c–f).

### In vivo role of NOX4 in anoikis resistance and distant metastasis

**Fig. 7:**
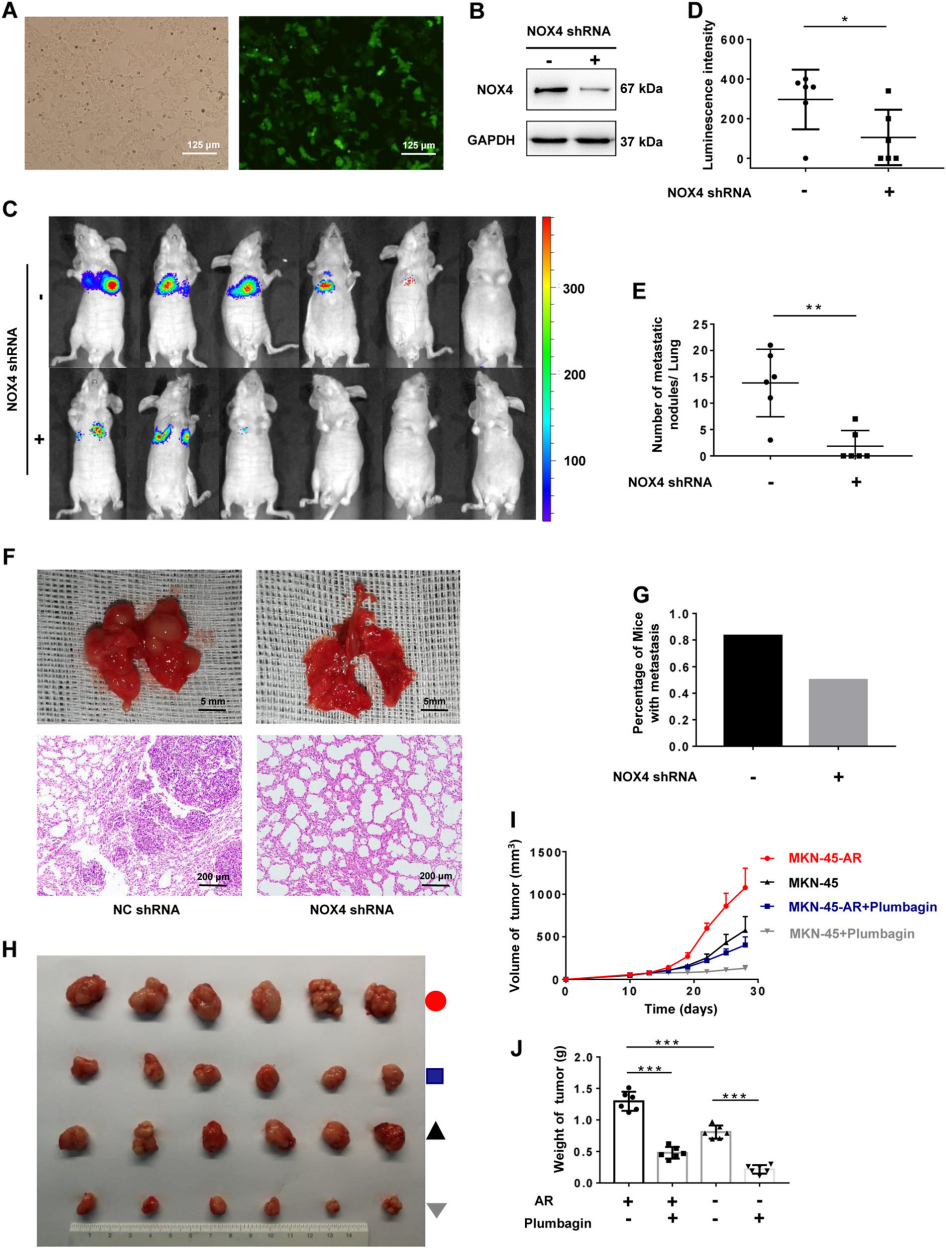
In vivo role of NOX4 in anoikis resistance and distant metastasis. Data from tail vein injection model were shown in panels (**a**–**g**) and data from xenograft model were shown in panels (**h**–**j**). **a** Verifying of infection efficiency by fluorescence (magnification ×200). **b** Validation of knockdown efficiency of shRNA-NOX4 using western blot. **c**, **d** MKN-45-luciferase cells with or without NOX4 knockdown were tail vein injected into nude mice. **c** Bioluminescence imaging was performed with an IVIS Lumina XR at week 8. **d** Average luminescence intensity of each group at week 8. *P < 0.05. **e** Number of metastatic nodules in lungs of mice in group-shRNA-NOX4 and group-shRNA-NC. **P < 0.01. **f** Representative image of lung of mice in group-shRNA-NOX4 and group-shRNA-NC. Bars represent 5 mm for morphology and 200 μm for H&E staining. **g** Percentage of mice with metastasis in group-shRNA-NOX4 and group-shRNA-NC. **h** Image of tumors of mice in group-MKN45-AR, group-MKN45-AR + Plumbagin, group-MKN45 and group-MKN45 + Plumbagin. **i** Volume of tumor of mice in group-MKN45-AR, group-MKN45-AR + Plumbagin, group-MKN45 and group-MKN45 + Plumbagin at indicated time. **j** Weight of tumor of mice in group-MKN45-AR, group-MKN45-AR + Plumbagin, group-MKN45 and group-MKN45 + Plumbagin on 28th days. ***P < 0.001

To assess the in vivo effects of NOX4 on anoikis resistance in peripheral blood circulation and distant metastasis, a mouse tumor model was established. MKN-45 cells were infected with lentivirus expressing shRNA-NOX4 or shRNA-NC and luciferase. The efficiency of infection was verified by fluorescence (Fig. 7a). Knockdown efficiency was validated at the levels of translation (Fig. 7b). A total of 10^6^ MKN-45 or MKN-45^**NOX4-KD**^ cells were injected into the tail vein of BALB/C nude mice and bioluminescence imaging performed with an IVIS Lumina XR. MKN-45 or MKN-45^**NOX4-KD**^ cells were traced in vivo at indicated time. The mice transplanted with NOX4 knockdown MKN-45 cells showed reduced metastasis compared with the group injected with MKN-45 cells and luminescence is mainly concentrated in the chest rather than upper abdomen (Fig. 7c, d). Eight weeks after injection, mice were sacrificed. The number of potential metastases on the surface of livers or lungs was counted, followed by pathological examination. None significant liver metastases appear in both groups. However, number of metastatic nodules in lungs of mice in group-shRNA-NOX4 is significantly less than those in group-shRNA-NC (Fig. 7e). Representative specimen or H&E staining images of lung of mice in group-shRNA-NOX4 and group-shRNA-NC are shown (Fig. 7f). Percentage of mice with metastasis in group-shRNA-NOX4 is lower than that in group-shRNA-NC (Fig. 7g). Above all, NOX4 depletion effectively suppressed the distant metastasis of GC, underlining a key role for NOX4 in anoikis resistance and the subsequent distant metastasis of GC.

To further explore the malignancy of anoikis-resistant GC versus nonresistant GC, as well as the value of NOX4-targeted therapy, we created a xenograft model that was established by subcutaneously injecting mice with MKN-45 or MKN-45^**AR**^ cells. Ten days after subcutaneous injection, the MKN-45 and MKN-45^**AR**^ groups were both mainlined with the specific NOX4 inhibitor plumbagin^24,25^ or normal saline. Tumor volumes were measured every 3 days. Twenty-eight days after subcutaneous injection, mice were sacrificed, and the weight and volume of tumors measured. The growth rate, volume, and weight of tumors in the MKN-45^**AR**^ group were much higher than the MKN-45 group and was decreased by plumbagin therapy (Fig. 7h–j).

Overall, the malignancy of anoikis-resistant GC was much higher than nonresistant GC, indicating that anoikis resistance contributes to the distant metastasis of GC. Furthermore, NOX4-targeted therapy shows potential as a strategy for inhibiting GC progression.

### NOX4 expression is positively correlated with EGFR expression in gastric cancer patients

**Fig. 8:**
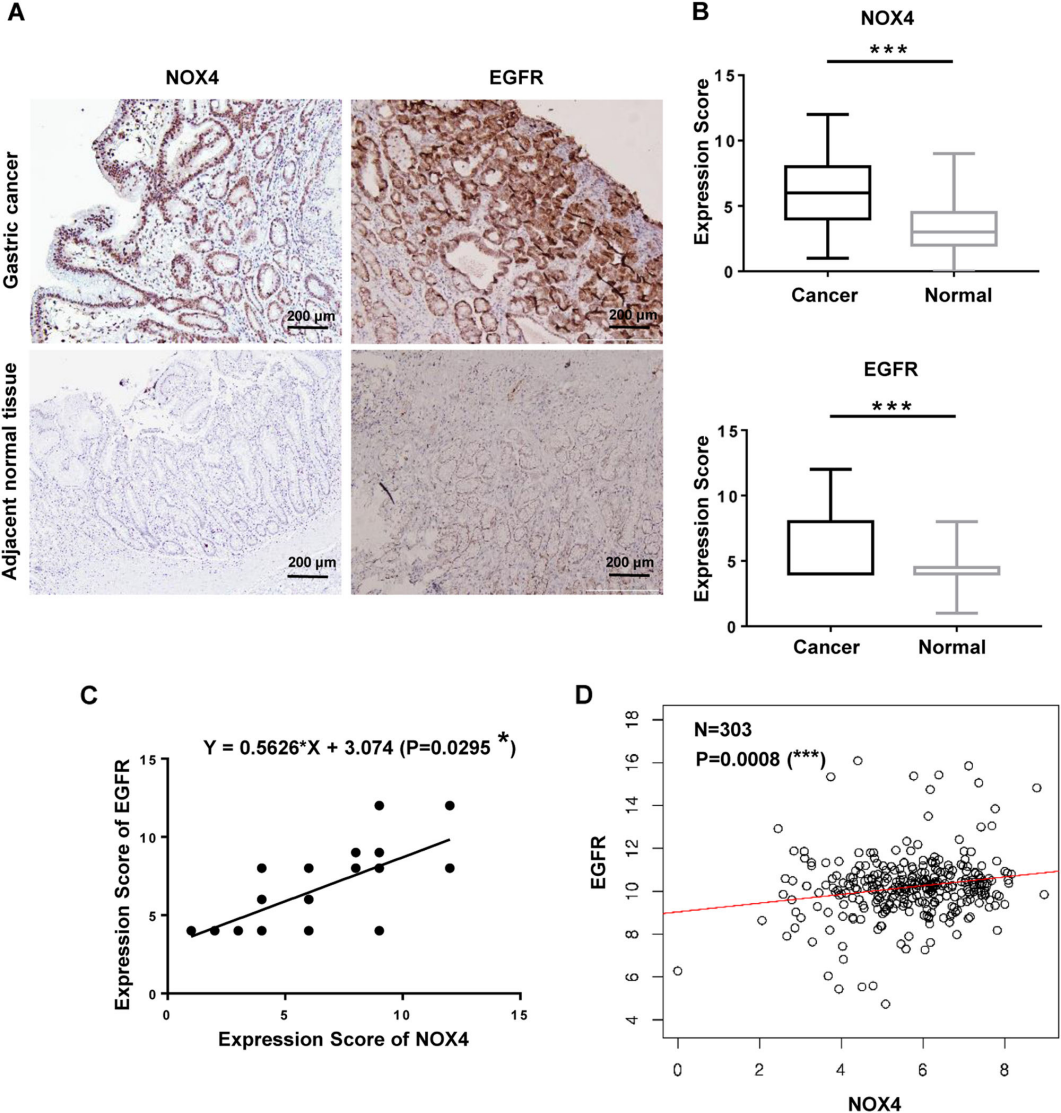
NOX4 expression is positively correlated with EGFR expression in gastric cancer. **a** Representative images of NOX4 and EGFR expression in gastric cancer tissues and adjacent normal tissues (magnification ×100). **b** Comparison of NOX4 and EGFR expression in gastric cancer tissues and paired adjacent normal tissues. ***P < 0.001. **c** Correlation between NOX4 and EGFR in gastric cancer tissues. *P < 0.05. **d** In silico analysis of correlation between NOX4 and EGFR in gastric cancer tissues using database (http://www.linkedomics.org/). ***P < 0.001.

To further explore the clinical correlation between NOX4 and EGFR expression in gastric cancer patients, we examined NOX4 expression in gastric cancer tissues and the paired adjacent normal tissues from 90 patients by IHC using anti-EGFR or anti-NOX4 antibodies (Fig. 8a, b). Interestingly, EGFR expression was increased in 68.42% (39/57) of NOX4-positive gastric cancer patients **(**Table 1). There was a significant positive linear correlation between NOX4 expression and EGFR expression (*P* **=** 0.0295) (Fig. 8c). Moreover, correlation analysis using database (http://www.linkedomics.org/) revealed a significant positive linear correlation between NOX4 expression and EGFR expression at mRNA level in gastric cancer (*P* = 0.0008) (Fig. 8d). These findings indicate that NOX4 expression contributes to the progression of gastric cancer and to metastasis, and this is likely associated with regulation of EGFR.

**Table 1:** Summary of NOX4 and EGFR expression in gastric cancer

Variables	Cases (*n*)	NOX4 expression		*P* value
		Low	High	
Low	39	21	18	0.0031**
High	51	12	39	

## Discussion

Metastasis plays a pivotal role in cancer-related deaths, including those caused by GC^3^. Anoikis is a programmed death process triggered by detachment from the ECM to prevent the dissemination of mis-localized cells^5^. The acquisition of anoikis resistance enables the survival of tumor cells in the blood circulation or lymphatic system, which is vital to successful cancer metastasis. However, the molecular mechanism behind tumor anoikis resistance remains elusive.

The regulation of redox homeostasis is essential for the maintenance of cell metabolism, growth, and survival^30^. However, ROS have a dual nature that produces both beneficial and harmful effects on cells in several diseases, including cancer^31^. The increased ROS generation in cancer cells is involved in the molecular and biochemical regulation that is essential to the tumor initiation^32^, progression^33^, and chemotherapy resistance^34^. In this case, supplementation with antioxidants could act as a brake for the tumor progression and metastatic potential^35^. However, escalated ROS may provide a unique access to eliminating cancer cells through elevating intracellular ROS to highly toxic levels, in turn, promoting ROS-induced cell death pathways^36^. In this case, supplementation with antioxidants or stimulation of antioxidant pathways actually promotes cancer cells survival by decreasing ROS-associated cytotoxicity^37,38^. In this sense, the dual effects of antioxidants in gastric cancer are not an exception^39,40^. Similarly, there are studies showing the different effects of redox regulation on anoikis resistance^41–43^. However, there is very little quantitative information on ROS levels (moderate or high levels) and their effect on the biological characteristics of cancer cells^30^. Our data revealed that detachment from the ECM elevated ROS levels moderately, which should be below the cytotoxic threshold. Supplementation with NAC, a ROS scavenger, increased the rate of anoikis of GC suspension cells. On the other hand, supplementation of H_2_O_2_ in small doses inhibited anoikis of GC suspension cells, which is in agreement with reports that lower concentrations of ROS are essential signaling molecules involved in cellular apoptosis, proliferation, and migration^6,7^. However, as the H_2_O_2_ doses increased, supplementation of H_2_O_2_ markedly promoted GC anoikis. These results indicate that the metabolic demands of detached cancer cells might differ depending on the cancer type and different metabolic conditions such as oxidase or antioxidase activities. Therefore, moderate increases in ROS in GC accounted for the anoikis resistance of GC cells in suspension conditions. Antioxidant that targets the NOX4−ROS−EGFR axis which induced anoikis resistance acts as an effective brake for the proliferation and metastatic progression of gastric cancer.

As major source of ROS in cancer, NADPH oxidases regulate redox-related signaling pathways^10^. It was demonstrated that NOX4 might play different roles in different types of cancers^12,14^. When it comes to gastric cancer, all three studies related to NOX4 and gastric cancer displayed a tumor promoter role of NOX4^44–46^. Similarly, our data revealed an obvious increase in NOX4 and NOX5 expression induced by detachment. NOX4 depletion in GC cells resulted in an obvious decrease in cell aggregation and increased rate of anoikis, whereas NOX5 depletion had little effect on cell aggregation or anoikis. Considering that NOX4 and DUOX1-2 generate H_2_O_2_ while NOX1-3 and NOX5 produces O_2_•−^47^, it is likely that the NOX4-derived H_2_O_2_ plays a crucial role in the anoikis resistance of GC. Furthermore, H_2_O_2_ induced NOX4-derived ROS signaling activation which, in turn, promotes cancer progression by inducing EMT, inhibiting apoptosis or promoting cell invasion^48–50^. Therefore, NOX4 might be constitutively active upon detachment from the ECM, likely forming a self-perpetuating loop in gastric cancer cells.

Redox metabolism plays a crucial role in the regulation of growth factor receptors-associated signaling pathway^51–53^. The maintenance of several growth factor receptor expression levels is essential for the induction of anoikis resistance^26,28,54–58^. In this study, silencing of NOX4 decreased ROS generation and subsequently downregulated EGFR expression, thereby sensitizing cells to anoikis. However, NOX4 regulation had little effect on other growth factor receptors. NOX4 upregulation attenuated downregulation of EGFR, inducing anoikis resistance. NOX4-derived ROS could directly regulate the expression of EGFR, in turn, promoting anoikis resistance of GC cells. It is likely that NOX4-derived ROS could regulate the transcription factor which could bind with promoter of EGFR, thereby, regulating EGFR expression. On the other hand, in view of the fact that anoikis induction requires the lysosomal-mediated downregulation of EGFR leading to the inhibition of prosurvival signaling^27,58^. Therefore, it is also likely that NOX4-derived ROS could suppress the trafficking and degradation of EGFR following cell detachment which could further promote anoikis resistance. Besides, it was demonstrated that EGFR was a receptor tyrosine kinase whose activation played fundamental roles in regulation of cell proliferation, differentiation, and survival^59^. However, the expression of p-EGFR in suspension GC was very weak. What’s more, either depletion or overexpression of NOX4 could hardly regulate p-EGFR levels in suspension GC. It is worth noting that EGFR has been demonstrated to exert some of its function independent of its ligand kinase or activation^60,61^. And in human cancer cells, kinase-independent EGFR could promote autophagic cell survival^62^. Besides, maintenance or stabilization of EGFR expression is an important mechanism through which cancer cells could evade anoikis^19,58^. It is conceivable that NOX4 promotes anoikis resistance through ROS-dependent upregulation of EGFR, which is independent of the activation of the EGFR in terms of phosphorylation.

The phenotypic and mechanistic controls that drive anoikis resistance permit the acquisition of migratory and invasive properties in cancer cells, leading to metastatic colonization^5,63–65^. Our data revealed that anoikis-resistant GC cells acquired stronger proliferative and invasive properties compared with nonresistant gastric cancer cells, and these properties were attenuated by NOX4 depletion. Intriguingly, GC^AR^ cells displayed larger and branched out invadopodia, which is associated with advanced invasion and migration of cancer cells^29^. Not surprisingly, silencing of NOX4 efficaciously blocked the distant metastasis of GC and therapy using plumbagin, a specific inhibitor of NOX4, inhibited the progression of GC in tumor-bearing mice, which was in accordance with the in vitro findings. Moreover, NOX4 expression is positively correlated with EGFR expression, which might convert them as promising biomarkers of gastric cancer for the clinics. In addition, overexpression or mutation of EGFR is involved in the malignant biological behavior of GC^66,67^. NOX4 might emerge as an attractive target in the treatment of GC. Plumbagin, as an NOX4 inhibitor for GC therapy, showed promising efficacy in the treatment of GC in athymic mice xenograft models. It is conceivable that targeting the NOX4−ROS−EGFR axis is a promising strategy to inhibit human GC progression.

## Materials and methods

### Cell culture

The gastric epithelial cell line GES-1 and gastric cancer cell lines MKN-45 and AGS were purchased from American Type Culture Collection (Manassas, VA, USA) and maintained in RPMI 1640 medium (Gibco, Waltham, MA, USA) supplemented with 10% fetal bovine serum (FBS; Gibco, Waltham, MA, USA), 1% l-glutamine and penicillin/streptomycin (Gibco, Waltham, MA, USA) at 37 °C in 5% CO_2_. Medium was renewed every 3 days and cells were passaged upon reaching 70–90% confluence.

### Suspension culture

Ultra-low attachment six-well plates were purchased from Corning (Corning, NY, USA). A single cell suspension was formed by trypsinization, and cells were plated at 10^6^ cells/well. After a 24 h incubation, the cells were harvested and processed for flow cytometric analysis, mRNA analysis, and protein analysis.

### Cultivation of anoikis-resistant cells

A single cell suspension was formed by trypsinization, and cells were plated on ultra-low attachment six-well plates at a concentration of 10^6^ cells/well. Cells were passaged every 3 days. After continuous passage, anoikis-resistant cells were acquired for analysis of biological characteristics.

### Cell apoptosis analysis

Detection of apoptosis was carried out using the Annexin V–FITC Apoptosis Detection Kit II (KeyGEN BioTECH, Nanjing, Jiangsu, China). Cells were harvested after treatment and washed 2–3 times with phosphate buffered saline (PBS). The cells were resuspended in 500 µL 1× binding buffer, and 5 µL Annexin V-FITC and 5 µL PI were added. After a 15 min incubation in the dark at room temperature, the percentage of cells undergoing apoptosis was analyzed by flow cytometry (FCM).

### Detection of ROS

For intracellular ROS measurements, cells cultured under attachment or detachment conditions were incubated with 25 mM of carboxy-H2DCFDA (Sigma, St. Louis, MO, USA) for 30 min at 37 °C, harvested, and analyzed by FCM on a flow cytometer (Becton Dickinson Bioscience, San Jose, CA, USA).

### siRNA transfection assay

GC cells were transfected with 90 nM NOX4-siRNA, EGFR-siRNA or control siRNA from Shanghai GenePharma Co., Ltd (Shanghai, China) using Lipofectamine 2000 reagent (Thermo Fisher Scientific, Waltham, MA, USA) according to the manufacturer’s instructions. The knockdown efficiency was examined by western blot and RT-qPCR. Cells were collected after 48 h post-transfection for western blot, qRT-PCR, and FCM analysis.

### Plasmid transfection assay

GC cells were transfected with 2 µg of NOX4 or specific control plasmid from Shanghai GenePharma Co., Ltd (Shanghai, China) using Lipofectamine 2000 reagent in accordance with the manufacturer’s instructions. The expression efficiency of NOX4 was examined by western blot and RT-qPCR. Cells were collected at 48 h post-transfection for western blot qRT-PCR FCM experiments.

### Lentiviral transduction

The lentiviral vector containing gene-specific shRNAs against NOX4 and a control lentiviral vector encoding scrambled shRNA were purchased from Shanghai GenePharma Co., Ltd (Shanghai, China). Human GC cell lines were transduced with the lentiviral particles along with polybrene and were selected by puromycin (1 mg/mL) (Thermo Fisher Scientific, Waltham, MA, USA) for 2 weeks. The knockdown efficiency was examined by western blot and RT-qPCR.

### Western blot analysis

Total protein from cells was extracted by lysis and resolved by 8–12% SDS-PAGE. Samples were then electro-transferred onto a polyvinylidene difluoride (PVDF) membrane (Millipore, Boston, MA, USA). PVDF membranes were blocked with 5% BSA or skimmed milk. Target proteins were detected with antibodies against GAPDH (Abcam, ab9485, diluted 1:2500), NOX1 (Abcam, ab78016, diluted 1:1000), NOX2 (Abcam, ab31092, diluted 1:1000), NOX3 (Abcam, ab82708, diluted 1:2000), NOX4 (Abcam, ab133303, diluted 1:2000), NOX5 (Abcam, ab198213, diluted 1:2000), DUOX1 (Abcam, ab178534, diluted 1:5000), DUOX2 (Abcam, ab170308, diluted 1:500), VEGFR-1 (Abcam, ab32152, diluted 1:2000), VEGFR-2 (Abcam, ab11939, diluted 1:1000), VEGFR-3 (Abcam, ab27278, diluted 1:1000), EGFR (Abcam, ab52894, diluted 1:5000), p-EGFR (Abcam, ab40815, diluted 1:2000), PDGFR-α (Abcam, ab203491, diluted 1:1000), PDGFR-β (Abcam, ab32570, diluted 1:10000) and C-Met (Abcam, ab51067, diluted 1:5000) overnight at 2–8 °C, followed by incubation with specific horseradish peroxidase (HRP)-conjugated secondary antibodies (Abcam, ab6721, diluted 1:10,000) for 2 h at room temperature. Bands were visualized by chemiluminescence with enhanced chemiluminescent detection reagents (Millipore, Boston, MA, USA).

### Reverse transcription quantitative real-time polymerase chain reaction (RT-qPCR)

Total RNA extraction was carried out in accordance with the instructions. RNA samples were then reverse transcribed to cDNA with an RT-PCR Kit (Takara, Kyoto, Japan). The primers were designed and synthesized by Genepharma (Shanghai, China). Next, qRTPCR was performed with SYBR Green premix Ex Taq on an ABI ViiA 7 Dx RT-PCR instrument. Cycling conditions were as follows: 1 cycle at 95 °C for 3 min; 40 cycles at 95 °C for 5 s, 60 °C for 34 s; 1 cycle at 95 °C for 15 s, 60 °C for 1 min, and 95 °C for 15 s. Human β-actin served as an internal reference. Relative mRNA expression was calculated using the 2^−ΔΔCt^ method.

### Immunofluorescence (IF)

Cells on glass slides were fixed by neutral paraformaldehyde (4%) and permeabilized using PBS containing 0.01% Triton X-100 for 15 min. Next, cells were incubated with anti-EGFR antibody (Abcam, ab52894, diluted 1:200). Additionally, cells were incubated with 4′,6-diamidino-2-phenylindole (DAPI) as a nuclear counterstain. A confocal laser scanning microscope (TCS SP8 STED ×3, Leica, Wetzlar, Germany) was used for confocal microscopy.

### Cell invasion assay

Anoikis-resistant GC cells were transfected with siRNA-NOX4 or siRNA-control. GC cells not selected for resistance were transfected with siRNA-control. Transwell chambers (6.5 mm, Costar, Corning, NY, USA) with a polycarbonate membrane insert (8 µm pore size) were pre-coated with Matrigel. Then, 10^5^ cells suspended in RPMI 1640 medium free of FBS were transferred to the upper chamber, with RPMI 1640 medium containing 10% FBS placed in the lower chamber. After a 24 h incubation, noninvaded cells were removed by cotton swabs and cells that invaded to the lower surface were fixed by 4% paraformaldehyde for 30 min and stained by crystal violet. Six fields per chamber were blindly selected for counting.

### Cell proliferation assay

GC cells were incubated with 50 μM EdU (KeyGEN BioTECH, Nanjing, Jiangsu, China) for 6–8 h and were fixed using 4% paraformaldehyde (pH 7.4) for 20–30 min at room temperature. Then, GC cells were washed with PBS for 3 × 5 min and then permeabilized with PBS containing 0.5% Triton X-100 for 20 min. The cells were washed with PBS extensively, and then were incubated with Apollo staining solution (KeyGEN BioTECH, Nanjing, Jiangsu, China) for 15–20 min. Next, the cells were washed using NaCl/Pi for 3 × 15 min and then incubated with DAPI (KeyGEN BioTECH, Nanjing, Jiangsu, China) for 15 min at room temperature.

### Scanning electron microscopy (SEM)

GC cells or anoikis-resistant GC cells were cultured on slides then fixed with PBS containing 2.5% glutaraldehyde overnight at 2–8 °C. Next, all samples were washed in PBS (3 × 10 min), followed by postfixation with PBS containing 1% OsO4 for 1 h at room temperature and rinsing with PBS (3 × 10 min). Then, the samples were dehydrated for 10–15 min at each step in a graded series of alcohol (30, 50, 70, 90, 95 and 100%), followed by dehydration with a mixture of isoamyl acetate and alcohol (1:1) for 30 min and isoamyl acetate (100%) for 60 min. Critical point drying was performed with liquid CO_2_. Finally, samples were coated using gold palladium and observed by SEM on a JEOL JSM-IT100 SEM (JEOL, Tokyo, Japan).

### Xenograft assay and bioluminescence imaging

Four-week-old male nude mice were injected intravenously with 10^6^ of MKN-45-Luciferase cells or MKN-45-Luciferase cells with NOX4 knocked down (MKN-45-Luciferase-NOX4-KD) through the tail vein. Mice were injected intraperitoneally with d-luciferin (30 mg/kg) 15 min before bioluminescence imaging. Images were acquired with an IVIS Lumina XR (Caliper, USA). Lastly, mice were sacrificed after two months and livers and lungs were harvested.

MKN-45 cells or anoikis-resistant MKN-45 cells were injected subcutaneously into nude mice. Seven days later, plumbagin (Thermo Fisher Scientific, Waltham, MA, USA, 5 mg/kg) or normal saline were injected into nude mice through the tail vein every 3 days. Tumor volumes (length × width^2^/2) were measured every 3 days. Mice were sacrificed after seven courses of treatment and tumors were harvested. Animal experiments were approved by the Animal Use and Care Committee of the Nanjing Drum Tower Hospital.

### Clinical tissue samples

Fresh GC tissues and adjacent normal tissues were obtained from patients who underwent resection of GC at the Nanjing Drum Tower Hospital of Nanjing University Medical School beginning in 2016. Patients who had received radiotherapy or chemotherapy before surgery were excluded. The clinical stages were confirmed according to the classification system of the American Joint Committee on Cancer (7th Edition). The study was performed in accordance with the guidelines of the Ethics Committee of the Nanjing Drum Tower Hospital of Nanjing University Medical School. Written informed consent was obtained from all patients.

### Immunohistochemical staining (IHC)

Tissues were fixed in formalin and embedded in paraffin, and then were cut into sections. The sections were deparaffinized using xylene and hydrated using alcohol, followed by antigen retrieval by the pressure-cooking method. The sections were incubated with primary NOX4 antibody (1:100 dilution, Abcam, Cambridge, UK) and EGFR (1:200 dilution, Abcam, Cambridge, UK) for 60 min at room temperature, followed by incubation with IgG H&L (HRP; 1:200 dilution, Abcam, Cambridge, UK). Then, the sections were stained with a chromogen and counterstained with hematoxylin. Scoring was comprehensively conducted depending on the staining intensity (0 for no staining, 1 for weak staining, 2 for moderate staining and 3 for strong staining) and percentage of positively stained cells (0 for 0–5% of cells, 1 for 6–25% of cells, 2 for 26–50% of cells, 3 for 51–75% of cells and 4 for 76–100% of cells). The product of both grades was calculated as the final expression score.

### In silico analysis

To investigate the correlation between NOX4 and EGFR expression in gastric cancer, we performed correlation analysis using database (http://www.linkedomics.org/).

### Statistical analysis

Statistical analysis was performed with SPSS 22.0 (IBM). Results from more than three independent experiments are expressed as the mean ± SEM. Comparisons between two sets of data were analyzed using Student’s *t* test. Comparisons among three groups were analyzed using analysis of variance. Comparisons of count data were performed using a chi-square test. The correlation between two variables was analyzed by linear-regression analysis. The differences were statistically significant when *P* < 0.05.

## Electronic supplementary material

Supplementary figure legend

Supplementary Figure 1

Supplementary Figure 2

Supplementary Figure 3

Supplementary Figure 4

Supplementary Table 1
