# Correction to: NADPH oxidase 4 regulates anoikis resistance of gastric cancer cells through the generation of reactive oxygen species and the induction of EGFR

**DOI:** 10.1038/s41419-025-07918-0

**Published:** 2025-10-28

**Authors:** Shangce Du, Ji Miao, Zhouting Zhu, En Xu, Linsen Shi, Shichao Ai, Feng Wang, Xing Kang, Hong Chen, Xiaofeng Lu, Wenxian Guan, Xuefeng Xia

**Affiliations:** 1 Department of General Surgery, Drum Tower Clinical Medical College of Nanjing Medical University, 321 Zhongshan Road, 210008 Nanjing, Jiangsu P. R. China; 2 Department of General Surgery, Nanjing Drum Tower Hospital, The Affiliated Hospital of Nanjing University Medical School, 321 Zhongshan Road, 210008 Nanjing, Jiangsu P. R. China

Correction to: *Cell Death & Disease* 10.1038/s41419-018-0953-7, published online 20 September 2018

During our subsequent research, we got some promising results for a new project proposal. Before going further, We carefully reviewed the data and found some errors that were inadvertently made during the assembly of the figure at the typesetting stage: Image of group-AGS in figure 6c were unintentionally misplaced.

The amended figure as well as the accompanying source data of the corrected group were summarized in the attachment and all the relevant raw-images were provided via the following link: https://onedrive.live.com/?id=D1DBAD3DE0FF4A7A%21s6f9bdd072f1248f6bed761a986491231&cid=D1DBAD3DE0FF4A7A.

**Original Fig. 6C** 
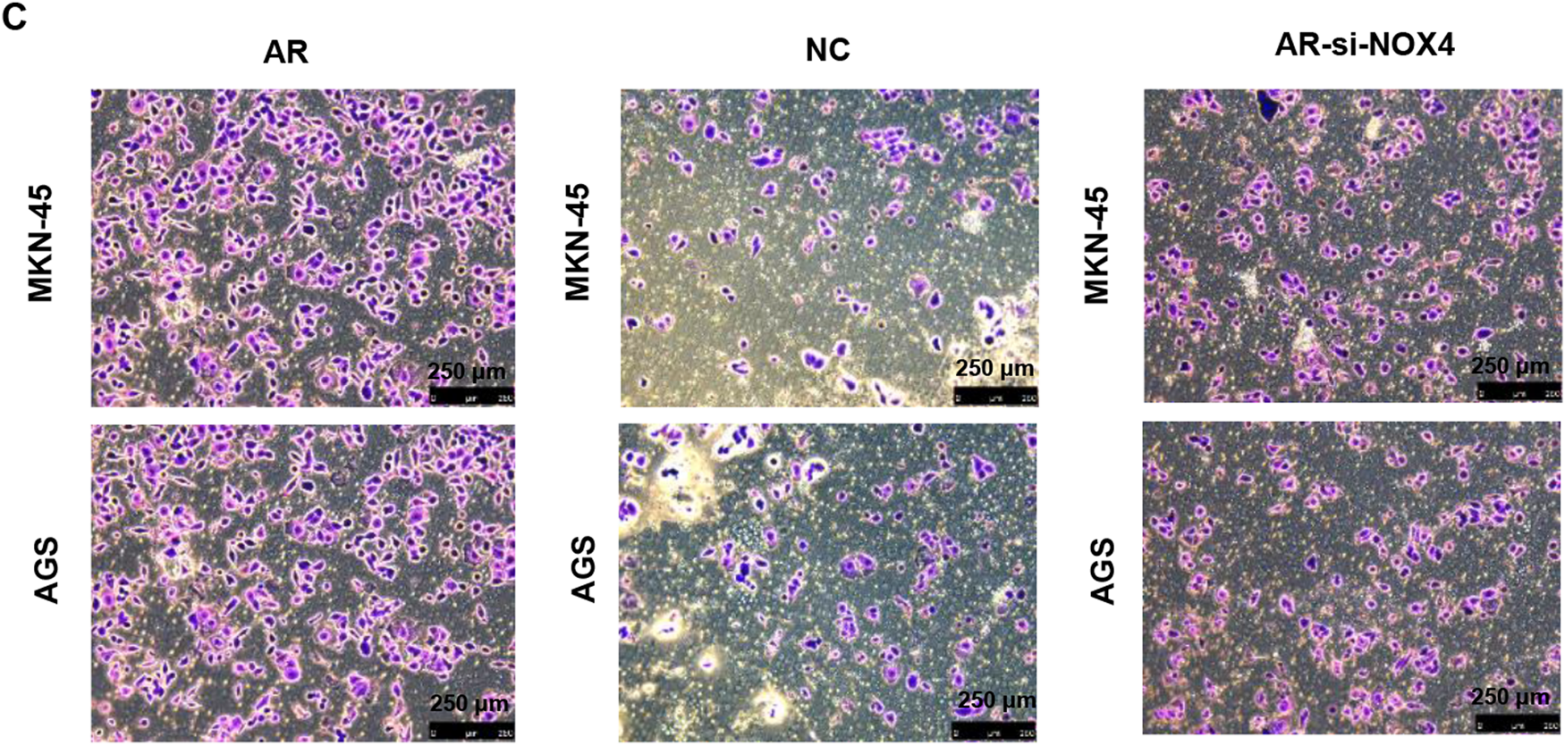


**Corrected Fig. 6C** 
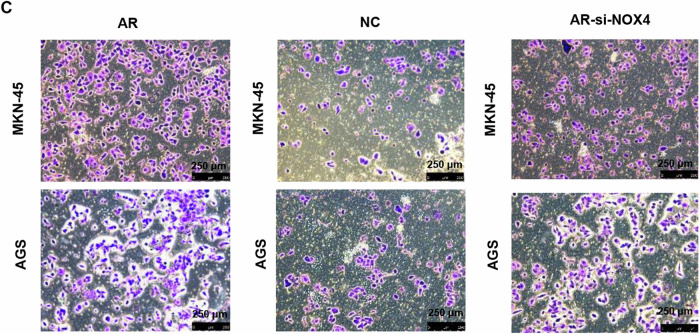


After double check, we could ensure that the change of representative image doesn’t influence the result and conclusion behind this panel.

