# Supplementary material for: NADPH oxidase 4 regulates anoikis resistance of gastric cancer cells through the generation of reactive oxygen species and the induction of EGFR
Source: Cell Death Dis. 2018 Sep 20;9(10):948. doi: 10.1038/s41419-018-0953-7 (PMC6148243; doi:10.1038/s41419-018-0953-7)
Supplement: Supplementary file 1 — Supplementary figure legend [file 41419_2018_953_MOESM1_ESM.docx]

**Supplementary figure legends**

**Supplementary Fig. 1.** Gastric cancer cells are more resistant to anoikis than normal gastric epithelial cells. (A) Number of GES-1, MKN-45 and AGS cells in suspension cultures on indicated days. (B) The rate of apoptosis of GES-1, MKN-45 and AGS cells cultured as adherent or suspension cultures for 24 h was detected by flow cytometry using Annexin V-FITC. **P* < 0.05, *****P* < 0.0001. (C) The morphology of MKN-45 and AGS cells cultured in suspension conditions as captured by microphotography at indicated times (magnification x100). (D) The average number of aggregated GES-1, AGS and MKN-45 cells in each field was measured 6 h after suspension cultivation. ****P* < 0.001. (E) GES-1, AGS and MKN-45 cells were cultured in attached or suspended conditions for 24 h, then protein was extracted. The expression levels of EGFR and cleaved caspase-3 were detected by Western blot with the indicated antibodies. GAPDH served as a loading control. All experiments were performed in triplicate.

**Supplementary Fig. 2.** NOX4, and not NOX5, is involved in regulation of anoikis resistance. (A) Cellular morphology of MKN-45 and AGS suspension cells treated with plumbagin or CHCl_3_ as captured with microphotography following 24 h of culture (magnification x100). (B) Cellular morphology of MKN-45 and AGS cells transfected with siRNA-NOX5 or siRNA-Negative as captured with microphotography following 24 h of suspension culture (magnification x100).

**Supplementary Fig. 3.** NOX4 regulates anoikis resistance and EGFR expression via ROS generation. MKN-45 and AGS cells were treated with plasmid-NC, Plasmid-NOX, Plasmid-NOX4 + NAC or Plasmid-NOX4 + H_2_O_2_ (1μM) (A-D). (A) The expression of NOX4 and EGFR was detected by immunoblotting with the indicated antibodies. (B) Anoikis rates of MKN-45 and AGS cells were measured by flow cytometry using Annexin V-FITC. ***P* < 0.01, ****P* < 0.001. (C) Cellular morphology of MKN-45 and AGS cells in suspension conditions captured by microphotography (magnification x100). (D) The average number of aggregated MKN-45 and AGS suspension cells in each field was measured after 24 h of culturing. **P* < 0.05; ***P* < 0.01.

**Supplementary Fig. 4.** NOX4 regulates anoikis resistance and EGFR expression via ROS generation. Negative control or NOX4-overexpressing MKN-45 and AGS cells were transfected with siRNA-Negative control or si-RNA EGFR for 24 h (A-D). (A) The expression of NOX4 and EGFR was detected by immunoblotting with the indicated antibodies. (B) The rate of anoikis of MKN-45 and AGS cells was measured by flow cytometry using Annexin V-FITC. ***P* < 0.01, ****P* < 0.001. (C) Cellular morphology of MKN-45 and AGS suspension cells captured by microphotography (magnification x100). (D) The average number of aggregated MKN-45 and AGS suspension cells in each field was measured after 24 h of culturing. **P* < 0.05; ****P* < 0.001. All experiments were performed three independent times.
