# Supplementary figures and images for: NADPH oxidase 4 regulates anoikis resistance of gastric cancer cells through the generation of reactive oxygen species and the induction of EGFR
Source: Cell Death Dis. 2018 Sep 20;9(10):948. doi: 10.1038/s41419-018-0953-7 (PMC6148243; doi:10.1038/s41419-018-0953-7)

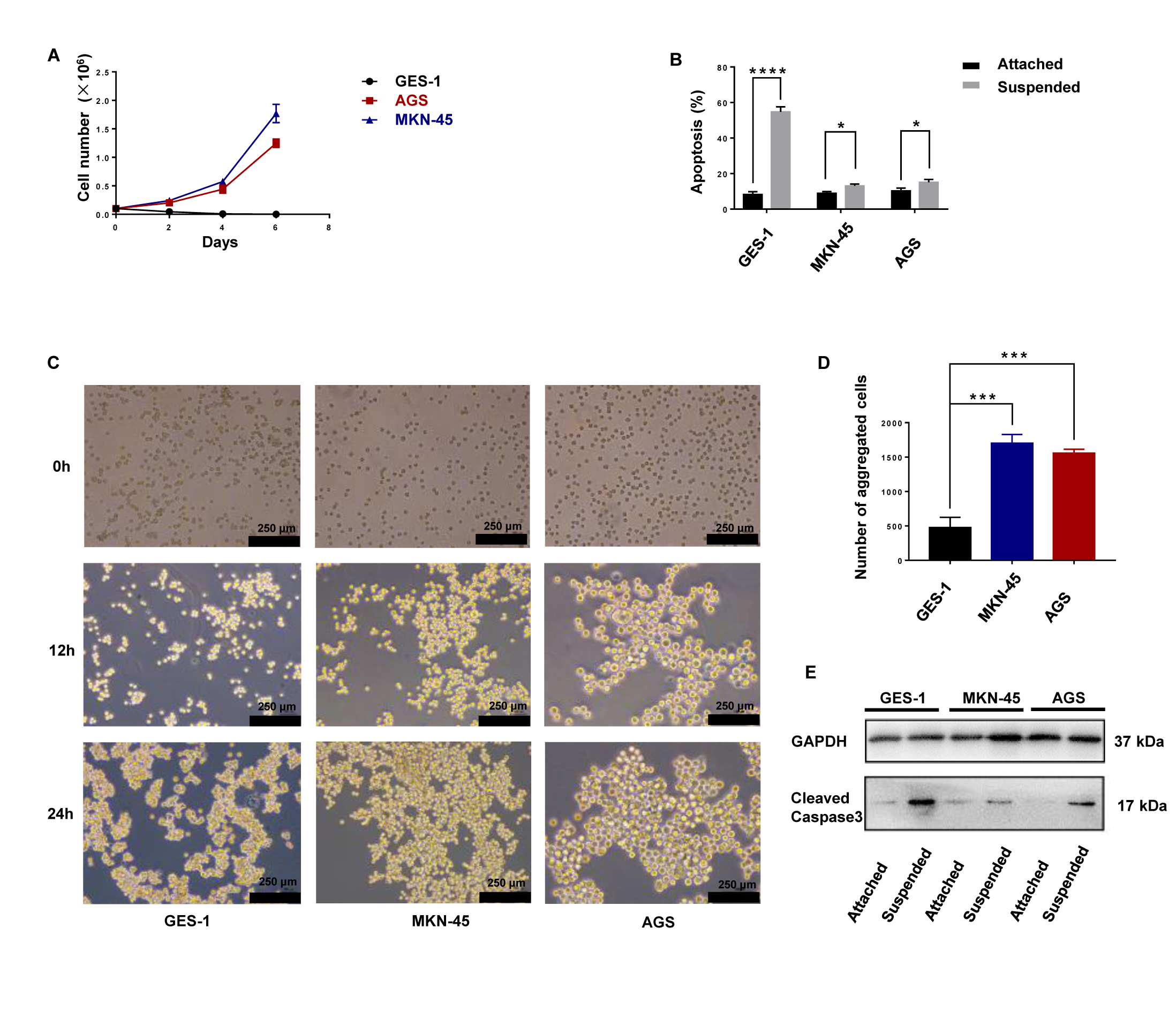

Supplement: Supplementary file 2 — Supplementary Figure 1 [file 41419_2018_953_MOESM2_ESM.tif]

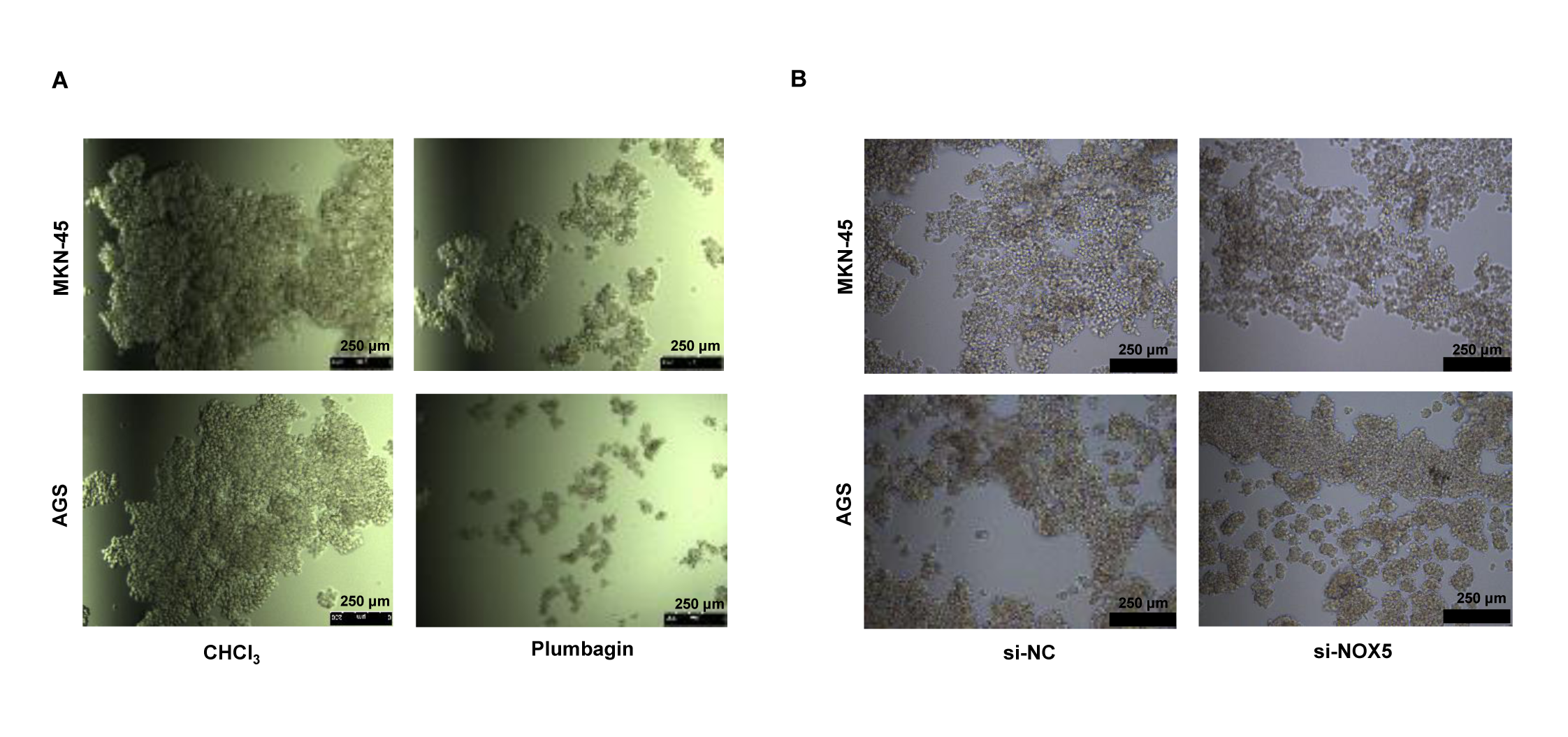

Supplement: Supplementary file 3 — Supplementary Figure 2 [file 41419_2018_953_MOESM3_ESM.tif]

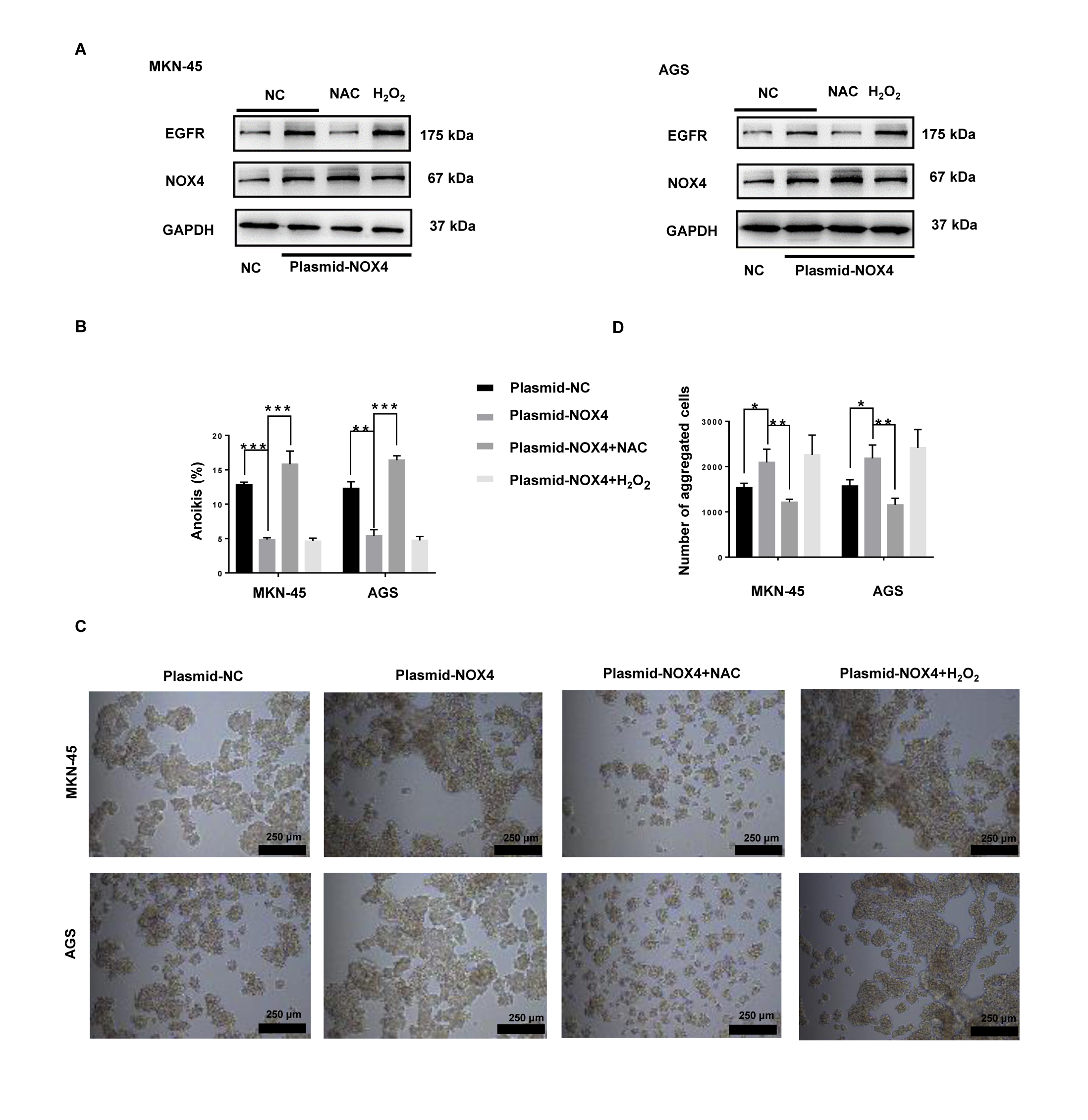

Supplement: Supplementary file 4 — Supplementary Figure 3 [file 41419_2018_953_MOESM4_ESM.tif]

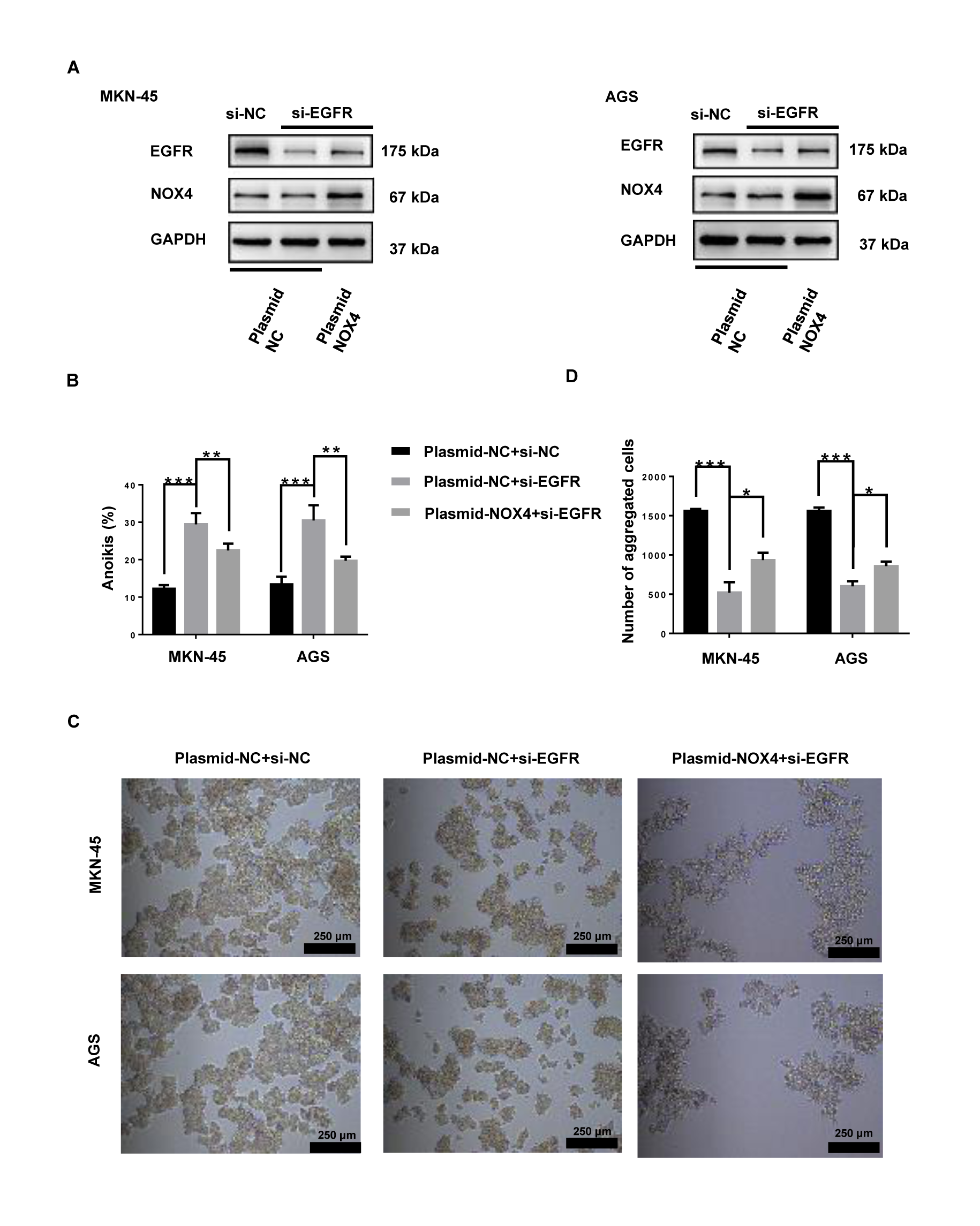

Supplement: Supplementary file 5 — Supplementary Figure 4 [file 41419_2018_953_MOESM5_ESM.tif]
