# Supplementary material for: NADPH oxidase 4 regulates anoikis resistance of gastric cancer cells through the generation of reactive oxygen species and the induction of EGFR
Source: Cell Death Dis. 2018 Sep 20;9(10):948. doi: 10.1038/s41419-018-0953-7 (PMC6148243; doi:10.1038/s41419-018-0953-7)
Supplement: Supplementary file 6 — Supplementary Table 1 [file 41419_2018_953_MOESM6_ESM.docx]

Table S1. Summary of primers used on the ABI ViiA 7 Dx RT-PCR instrument.

| **Gene** | **Species** | **Direction** | **Sequence (5’ to 3’)** |
| --- | --- | --- | --- |
| GAPDH | Human | F | AGCCACATCGCTCAGACAC |
|  |  | R | GCCCAATACGACCAAATC |
| NOX1 | Human | F | GGGGTCAAACAGAGGAGAGC |
|  |  | R | CTTCTGCTGGGAGCGGTAAA |
| NOX2 | Human | F | GAATGGTGTGTGAATGCCCG |
|  |  | R | ACAGCGTGATGACAACTCCA |
| NOX3 | Human | F | ACCGTGGAGGAGGCAATTAG |
|  |  | R | AGTGGTAGCGTTCCAGGTTG |
| NOX4 | Human | F | AGGAGAACCAGGAGATTGTTG |
|  |  | R | GGGATGACTT ATGACCGAAAT |
| NOX5 | Human | F | CAGCTTATGGGCTACGTGGT |
|  |  | R | GAACCGTGTACCCAGCCAAT |
| DUOX1 | Human | F | TCGTGACTATTTTGAGGGCAGT |
|  |  | R | GAGCCGGGCAACAATCCA |
| DUOX2 | Human | F | ATCCGAAATACCACCCTGCG |
|  |  | R | AAGTCAAGCACAGTCAGGGG |
| EGFR | Human | F | GCAAATAAACCGGACTGAAG |
|  |  | R | GTGGCACCAAAGCTGTATTTG |
| C-Met | Human | F | TACCCCAGCCCAAACCATTT |
|  |  | R | TGTGCTCCCACCACTAATAAAAGA |
| VEGFR-1 | Human | F | CTGGGCAGCAGACAAATCCT |
|  |  | R | ATTGCCATGCGCTGAGTGAT |
| VEGFR-2 | Human | F | AATCTCTGGTGGAAGCCACG |
|  |  | R | AATCGTCAGTACATGCCCCG |
| VEGFR-3 | Human | F | CACCGTGTGGGCTGAGTTTA |
|  |  | R | TGACGTTGTGGATGGTCAGG |
| PDGFR-α | Human | F | AGCACCTTCGTTCTGACCTG |
|  |  | R | TATTCTCCCGTGTCTAGCCCA |
| PDGFR-β | Human | F | TGATGCCGAGGAACTATTCATCT |
|  |  | R | TTTCTTCTCGTGCAGTGTCAC |
